# Is thymocyte development functional in the aged?

**DOI:** 10.18632/aging.100027

**Published:** 2009-02-17

**Authors:** Danielle Aw, Alberto B. Silva, Donald B. Palmer

**Affiliations:** Infection & Immunity and Genes & Development Group, Department of Veterinary Basic Sciences, Royal Veterinary College, UK

**Keywords:** aging, thymocyte, thymus, immunity, T cells

## Abstract

T cells are an integral part of a functional immune system with the majority being produced in the thymus. Of all the changes related to immunosenescence, regression of the thymus is considered one of the most universally recognised alterations. Despite the reduction of thymic size, there is evidence to suggest that T cell output is still present into old age, albeit much diminished; leading to the assumption that thymocyte development is normal. However, current data suggests that recent thymic emigrant from the aged thymus are functionally less responsive, giving rise to the possibility that the generation of naïve T cell may be intrinsically impaired in the elderly. In light of these findings we discuss the evidence that suggest aged T cells may be flawed even before exiting to the periphery and could contribute to the age-associated decline in immune function.

## The role of thymocyte development in T cell immunosenescence

One of the most universally recognised changes of the ageing immune system is the dramatic regression of the thymus; which in part is responsible for the observed clinical features of immunosenescence [1,2,3]. The features of age-related thymic atrophy involve a reduction in tissue mass, loss of tissue structure and abnormal architecture and a decline in thymocyte numbers leading to a reduction in naïve T cell output [3,4,5]. Despite the decline in the number of T cells exiting the thymus [6,7], there are no discernable changes in the number of T cells in the periphery with age [8], which appears to be tightly regulated by homeostatic mechanisms [9,10]. However, with increasing age peripheral T cells exhibit altered phenotypes, loss of diversity and modifications in responses, which have been correlated to shortened telomere and is related to replicative senescence [11,12,13]. These changes are (in part) a consequence of reduced naïve T cell output; however new evidence has revealed that recent thymic emigrants (RTE) from  the aged thymus exhibit reduced proliferative and functional activity [7,14,15]; thereby further contributing to T cell senescence. Specifically, aged RTE undergo phenotypic maturation with delayed kinetics [7] and exhibit a decreased proliferative capacity and a weak expression of early activation markers together with a lower production of IL-2 [7]. Furthermore, aged RTE are defective in increasing intracellular calcium concentration following TCR crosslinking [14] and exhibit reduced helper and memory activity [15].  Moreover, these studies also question the notion regarding whether T cell development is functionally active in the aged thymus; which is often assumed. This is largely based on the observations that there are no age-related differences in the proportion of the major subpopulations of thymocytes either in mice [16] or humans [17] and that T cell output can still be detected in the aged thymus [6,7]. This frequently leads to the belief that there is only a quantitative decline but no age-associated qualitative changes in thymopoiesis. However, with these recent studies showing intrinsic functional defects in aged RTE, it suggests that these newly generated T cells are already compromised prior to entry into the periphery indicating that various stages of differentiation are altered in an age-dependent manner.

## An overview of thymopoiesis

T cell development involves a series of sequential developmental steps requiring instructions from the specialised thymic microenvironment to regulate phases of proliferation, gene rearrangement and selection [18,19]. Each maturational stage is reflected by changes in gene and protein expression, which in turn is mirrored by modifications of cell surface markers, enabling the identification of thymocytes at various phases of development [20]. Briefly, thymocyte progenitors entering the thymus are identified by the absence of either co-receptor molecules CD4 and CD8 and are referred to as double negative (DN) thymocytes [20]. Within this subset several critical events occur, including commitment to the T cell lineage and cellular proliferation [21]. Subsequently, thymocytes become double positive (DP) for the expression of CD4 and CD8 with further maturation dependent on proceeding past positive and negative selection. Positive and negative selection facilitates the generation of functionally responsive and self-tolerant T cells [22,23], whereby DP thymocytes then mature into either single positive (SP) CD4^+^ T helper cells or SP CD8^+^ cytotoxic T lymphocytes before being exported into the periphery [24].

## The effect of age on the phenotype and function of developing thymocytes

Whilst it is widely acknowledged that there is a decline in the frequency and absolute number and precursor activity of early thymic progenitors (ETP) in older mice [1,25,26,27], it is often attributed to alterations in haematopoietic stem cells [28,29]. However, there is increasing evidence to suggest that the defects in ETP are due to cell intrinsic deficits that arise from exposure to an ageing thymic microenvironment [25,26,30,31]. For instance, there is an increase in the frequency of ageing ETP undergoing apoptosis in older mice [25,26] which is accompanied by a significant reduction in frequency of Ki67^+^ ETP in the aged thymus [25]; therefore these observations may account for the reduction in ETP number with age. Furthermore, these properties of ETP appear to be governed by signals derived from the thymus. Intravenously injected lineage negative-enriched bone marrow from young mice into sublethally irradiated one month old and 18 month old mice showed absolute number of donor cells was similar in young and older hosts after three days [31]. However, seven to ten days after injection, the number of donor cells in older thymi was severely reduced compared to those identified in younger thymi, suggesting a decline in their proliferative capacity [31]. In addition, when fetal thymi were grafted onto the kidney capsule of young and old mice, the thymic grafts had similar total thymic cellularity despite the native thymus from older animals still having significantly lower actual and subset numbers [30], suggesting the age-associated alterations in ETP is related to intrathymic changes. Moreover, it would not be unreasonable to assume that the defects that arise in the aged ETP, could also lead to the acquisition of further aberrations throughout thymopoiesis.

**Figure 1.**
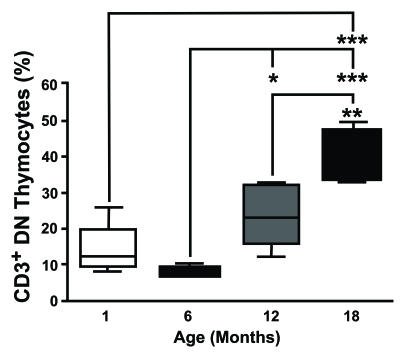
CD3 expression on DN thymocytes shows an age-dependent increase. Thymocytes from different aged mice were stained with anti-CD3, anti-CD4 and anti-CD8 mAb, analysed by flow cytometry and CD3 on DN cells was determined gating the appropriate population. This study revealed that the proportion of CD3^+^ DN thymocytes showed an age-dependent increase.  (One month n=5; six months n=5; 12 months n=8; 18 months n=4). *P<0.05; **P<0.01; ***P<0.001.

ETP are contained within the earliest stages of the DN subset and various reports have proposed several changes within this subpopulation; however, the results have not been consistent. Some groups have observed an increase only in the proportion of DN1 thymocytes but not other significant changes [30], while others have depicted an increase in DN1 and a subsequent decrease in DN3 subset [32]. In contrast, different laboratories have described an increase at the DN3 stage and a decrease in DN4 thymocytes [16], whilst no significant differences have been reported in percentage of DN thymocytes by other groups [33]. These discrepancies could arise from the different strains of mice analysed and the timepoints examined. Nevertheless, there is data to indicate that the DN subpopulation is subject to phenotypical and functional alterations with age. Interestingly a number of groups, including our own (Figure 1), have observed an increase in the expression of CD3, the signalling transduction complex of the T-cell receptor (TCR), within the DN compartment [34]. Corresponding to CD3 upregulation, these cells appear to express high levels of CD44 [34]. Previously a population of CD44^+^CD24^-^CD3^+^ DN cells has been described, which accumulates in older mice, and it has been suggested that these cells belong to a separate lineage [35]; perhaps representing NK1.1^+^ thymocytes, which display a similar phenotype [36]. Interestingly, a similar population has been identified in adult murine bone marrow and have been associated with a role in downregulation of haematopoiesis [37]. Therefore, this expanding population may not only represent an alternate lineage but may have deleterious affects on developing thymocytes.

**Figure 2.**
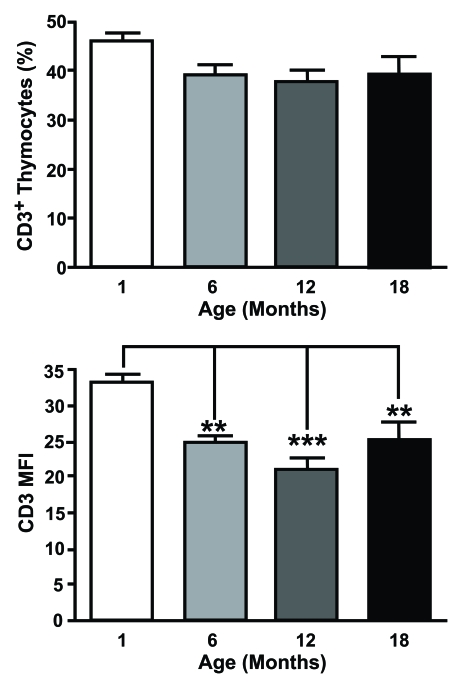
CD3 expression is altered on aged thymocytes. Thymocytes from different aged mice were stained with anti-CD3 mAb and analysed by flow cytometry. The top histogram shows the percentage of CD3+ cells positive and the bottom shows mean fluorescent intensity (MFI) of CD3 expression for one month old, six month old, 12 month old and an 18 month old animals. MFI was obtained by gating on the entire population. Although there were no age-related changes in the proportion of CD3+ thymocytes, a significant decrease in the number of CD3 molecules on thymocytes associated with age was observed. (One month n=5; six months n=5; 12 months n=8; 18 months n=4). **P<0.01; ***P<0.001.

**Figure 3.**
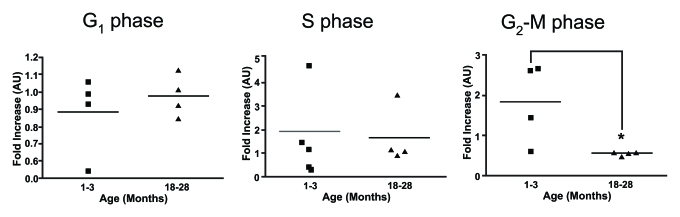
Cell cycle analysis on stimulated thymocytes from young and old mice. The various stages of the cell cycle in thymocytes from young and old mice following treatment with ConA and IL-2 after 24 hours was determined by flow cytometry. Data is expressed as fold increase compared to time zero. It was observed that there was a significant increase in the proportion of thymocytes from young mice at the G_2_-M phase compared to thymocytes from older animals. One month n=4; 18 months n=4. *P<0.05.

Despite an increase in the proportion of DN thymocytes expressing CD3, there is a declining trend in the percentage of CD3^+^ thymocytes from both humans [17] and mice [38]. This is accompanied by a significant decrease in CD3 median fluorescence index (MFI) on murine thymocytes with age, corresponding to the average number of complexes per cell (Figure 2). This alteration could have gross implications for the developing thymocytes. Considering that the CD3 complex is integral for relaying TCR signals [39], a decrease in the number of CD3 molecules would affect the ability of T cells to respond to such TCR-dependent signals and hence impair thymopoiesis [40]. Indeed, studies by Li and colleagues showed that murine thymocytes stimulated with ConA, which acts through the TCR, together with interleukin-2 (IL-2) displayed an age-related decline in proliferation as measured by trititated thymidine incorporation [16]. A similar finding was also observed using rat thymocytes [41]. Cell cycle analysis by propidium iodine conducted in our laboratory provides further support for a defect in the proliferative response to ConA and IL-2 by thymocytes from older mice with the results suggesting the deficiency is an inability to progress from S phase to the G2/M phase of the cell cycle (Figure 3). Although these studies suggest there is an impairment of TCR-expressing thymocytes to proliferate, it is unclear whether this reflects a shortcoming in all thymocyte populations or if this is related to the age-associated decrease in CD3 expression. However, an *in vivo* method to assess intrathymic proliferation in humans, employing T cell receptor excision circle (TREC) ratio analysis, implied that not all thymocyte populations undergo an age-dependent deficit to proliferate, and only thymocytes in later stages of maturation are affected [42]. This appears to correlate with the changes observed in RTE of older mice, which display a decline in proliferation and activation [7,14]. Therefore, the proliferative impairments observed in RTE from ageing mice could arise from intrinsic defects imprinted on the developing T cells in the thymus.

**Figure 4.**
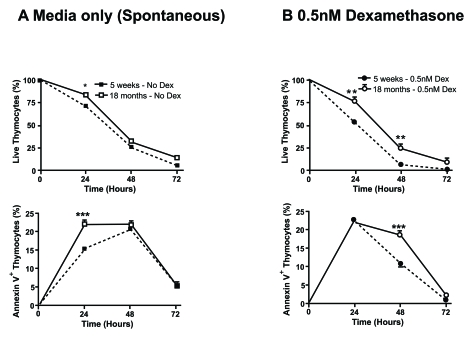
Aged thymocytes have increased resistance to spontaneous and dexamethasone-induced apoptosis. Spontaneous **(A)** and dexamethasone (dex)-induced **(B)** apoptosis at 0.5nM was assessed by flow cytometry. Graphs show the percentage of viable thymocytes defined as Annexin V^- ^7AAD^-^ (top graphs)and those undergoing early apoptosis as Annexin V^+ ^7AAD^-^ (bottom graphs). Closed square/circle with dotted line symbolise young thymocytes cultured in media or with the addition of 0.5nM dex respectively. Whereas, open square/circle with solid line signify thymocytes from 18 month old mice cultured in media or with the addition of 0.5nM dex respectively. The data revealed that there is an age-associated increased resistance to spontaneous and dex-induced apoptosis with a higher percentage of viable thymocytes from older mice compared to younger mice and delayed kinetic of older thymocytes to initiate apoptosis. Data representative of four experiments. *P<0.05; **P<0.01.

Aged peripheral T cells from either humans or mice demonstrate an increased resistance to apoptosis [43,44]. Although these cells may represent the most terminally differentiated T cells, suggesting that increased resistance to apoptosis is the outcome of senescence, a study investigating *in vivo* responses to activation induced cell death of T cells from aged mice implies age-related impairment of apoptosis can occur in previously unchallenged T cells and is perhaps intrinsically acquired [45]. In this study, male SCID mice receiving adoptively transferred T cells from old female HY TCR transgenic mice had a three-fold increase in the percentage of autoreactive CD8^+^ HY antigen-reactive T cells in contrast to mice receiving T cells from young female transgenic mice. Moreover, in our laboratory we have observed an age-dependent resistant to spontaneous and dexamethasone-induced apoptosis in murine thymocytes (Figure 4), which has also been reported in rat thymocytes [41].  Therefore, the resistance to apoptosis observed in thymocytes from older mice may be reflected in decreased susceptibility of peripheral T cells to undergo cell death.

Collectively these studies corroborate to argue for the occurrence of age-related deficiencies in T cell development that are similar to those seen in aged RTE and therefore the abnormalities observed in these cells are likely to have been acquired during thymopoiesis; primarily due to a defective microenvironment [15,30,31]. Thus, thymocytes may be defective before export into the periphery and could contribute to T cell immunosenescence. However, it is clear that this area warrants further investigation, including assessing the diversity of thymocyte receptors with age and evaluating the affect of ageing on selection.

## What is the significance of defective thymopoiesis in the elderly?

Considering these findings, the question then arises, what are the implications of defective thymopoiesis? Especially, given the significant decrease in T cell output by the thymus with age [6,7,46] and that maintenance of the peripheral T cell pool is believed to be predominantly maintained by homeostatic proliferation [9], how much can alterations in the properties of newly generated T cells in the elderly contribute to immunosenescence? The rate of daily export has been determined as 1-2% of the total thymocyte population [47] and is under control of mechanisms independent of the peripheral T cell pool. Furthermore, RTE are excluded from the niche-based regulation of peripheral T cell numbers [48] and are preferentially selected for survival in the periphery over existing resident T cells [47]. Therefore, the thymus is able to influence the T cell pool throughout adult life with considerable control over the composition of the peripheral T cell pool repertoire.

Since diversity in the elderly is dependant on the generation of RTE, defects in their development have as a profound affect on T cell immunosenescence as those acquired in the periphery. This has major implication for new and emerging diseases in the elderly, given the importance of these cells in immune protection. Moreover in light of these recent findings, methods that are designed to increase thymic output should also consider targeting the thymic microenvironment. Indeed, where successful strategies have reversed thymic involution in old mice, they appear to have done so by targeting the thymic microenvironment [32,49,50].

## Impact on the aged thymic microenvironment

The consequence of defective thymopoiesis may also have more local effects. Thymocytes and the thymic stroma exist in a bidirectional symbiotic relationship. Several experiments have now provided evidence that whilst initial patterning of the thymic epithelial compartment is thymocyte independent, maintenance and continued development requires the presence of differentiating T cells. Indeed, abrogation of thymopoiesis at different stages determines the severity and disruption of the thymic architecture [51,52,53,54]. In mice with defects affecting the later stages of thymocyte development concerning the DP to SP transition, the thymic medulla, which is the thymic niche responsible for ensuring tolerance and directing egression from the thymus, is absent [51,52]. Thymopoiesis blocked at earlier stages of development involving the DN compartment results in a loss of medulla and cortex, with the latter necessary to initiate T lineage commitment and provide signals for gene rearrangement and survival [53,54]. Furthermore, impairment of the bidirectional relationship between thymocytes and TEC causes alterations in thymic epithelial cell numbers [55]. The absence of either lymphotoxin β rector on thymic epithelial cells, its ligand on thymocytes or its intracellular signaling molecule nuclear factor-κB-inducing kinase, results in the disorganization of medullary thymic epithelial cells [55]. Therefore, considering the age-related alterations throughout T cell development, it may induce alterations in the thymic microenvironment. Indeed, we have found a decline in definitive thymic epithelial cell markers and disruption of the cortex and medulla [4], concurrent to alterations in the three dimensional structure [56]. It remains unclear whether changes in thymopoiesis are cause or effect of the altered thymic microenvironment, although recent data implies the thymic stroma might be the initiator [4,30,31]. Nevertheless, thymic involution could be exacerbated by the formation of a negative feedback loop with deterioration in the stromal compartment influencing a decline in thymocyte development, which in turn intensifies the changes in thymic epithelial cells.

## Concluding remarks

The qualitative contribution of newly generated T cells to the process of immunosenescence is often overlooked, despite alterations in their quantity being widely acknowledged. However, considering the evidence, we propose that in spite of continual T cell output from the thymus throughout life, the thymocytes from which they are derived are inherently defective and these shortcomings are acquired during thymopoiesis. Furthermore, we believe that these cells can significantly contribute to the age-associated changes observed in the periphery and exacerbate the alterations in thymocyte development through their interaction with the thymic microenvironment.

## References

[1] Aw D, Silva AB, Palmer DB (2007). Immunosenescence: emerging challenges for an ageing population. Immunology.

[2] Pawelec G, Akbar A, Caruso C, Solana R, Grubeck-Loebenstein B, Wikby A (2005). Human immunosenescence: is it infectious. Immunol Rev.

[3] Aspinall R, Andrew D (2000). Thymic atrophy in the mouse is a soluble problem of the thymic environment. Vaccine.

[4] Aw D, Silva AB, Maddick M, von Zglinicki T, Palmer DB (2008). Architectural changes in the thymus of aging mice. Aging Cell.

[5] George AJ, Ritter MA (1996). Thymic involution with ageing: obsolescence or good housekeeping. Immunol Today.

[6] Douek DC, McFarland RD, Keiser PH, Gage EA, Massey JM, Haynes BF, Polis MA, Haase AT, Feinberg MB, Sullivan JL, Jamieson BD, Zack JA, Picker LJ (1998). Changes in thymic function with age and during the treatment of HIV infection. Nature.

[7] Hale JS, Boursalian TE, Turk GL, Fink PJ (2006). Thymic output in aged mice. Proc Natl Acad Sci U S A.

[8] Hulstaert F, Hannet I, Deneys V, Munhyeshuli V, Reichert T, De Bruyere M, Strauss K (1994). Age-related changes in human blood lymphocyte subpopulations. II. Varying kinetics of percentage and absolute count measurements. Clin Immunol Immunopathol.

[9] Goronzy JJ, Weyand CM (2005). T cell development and receptor diversity during aging. Curr Opin Immunol.

[10] Akbar AN, Fletcher JM (2005). Memory T cell homeostasis and senescence during aging. Curr Opin Immunol.

[11] Plunkett FJ, Franzese O, Finney HM, Fletcher JM, Belaramani LL, Salmon M, Dokal I, Webster D, Lawson AD, Akbar AN (2007). The loss of telomerase activity in highly differentiated CD8+CD28-CD27- T cells is associated with decreased Akt (Ser473) phosphorylation. J Immunol.

[12] Haynes L, Swain SL (2006). Why aging T cells fail: implications for vaccination. Immunity.

[13] Nikolich-Zugich J (2005). T cell aging: naive but not young. J Exp Med.

[14] Clise-Dwyer K, Huston GE, Buck AL, Duso DK, Swain SL (2007). Environmental and intrinsic factors lead to antigen unresponsiveness in CD4(+) recent thymic emigrants from aged mice. J Immunol.

[15] Eaton SM, Maue AC, Swain SL, Haynes L (2008). Bone marrow precursor cells from aged mice generate CD4 T cells that function well in primary and memory responses. J Immunol.

[16] Li L, Hsu HC, Grizzle WE, Stockard CR, Ho KJ, Lott P, Yang PA, Zhang HG, Mountz JD (2003). Cellular mechanism of thymic involution. Scand J Immunol.

[17] Bertho JM, Demarquay C, Moulian N, Van Der Meeren A, Berrih-Aknin S, Gourmelon P (1997). Phenotypic and immunohistological analyses of the human adult thymus: evidence for an active thymus during adult life. Cell Immunol.

[18] Hayday AC, Pennington DJ (2007). Key factors in the organized chaos of early T cell development. Nat Immunol.

[19] Anderson G, Jenkinson EJ (2001). Lymphostromal interactions in thymic development and function. Nat Rev Immunol.

[20] Ceredig R, Rolink T (2002). A positive look at double-negative thymocytes. Nat Rev Immunol.

[21] Fehling HJ, von Boehmer H (1997). Early alpha beta T cell development in the thymus of normal and genetically altered mice. Curr Opin Immunol.

[22] von Boehmer H, Aifantis I, Gounari F, Azogui O, Haughn L, Apostolou I, Jaeckel E, Grassi F, Klein L (2003). Thymic selection revisited: how essential is it. Immunol Rev.

[23] Werlen G, Hausmann B, Naeher D, Palmer E (2003). Signaling life and death in the thymus: timing is everything. Science.

[24] Germain RN (2002). T-cell development and the CD4-CD8 lineage decision. Nat Rev Immunol.

[25] Min H, Montecino-Rodriguez E, Dorshkind K (2004). Reduction in the developmental potential of intrathymic T cell progenitors with age. J Immunol.

[26] Heng TS, Goldberg GL, Gray DH, Sutherland JS, Chidgey AP, Boyd RL (2005). Effects of castration on thymocyte development in two different models of thymic involution. J Immunol.

[27] Min H, Montecino-Rodriguez E, Dorshkind K (2005). Effects of aging on early B- and T-cell development. Immunol Rev.

[28] Tyan ML (1977). Age-related decrease in mouse T cell progenitors. J Immunol.

[29] Donnini A, Re F, Orlando F, Provinciali M (2007). Intrinsic and microenvironmental defects are involved in the age-related changes of Lin - c-kit+ hematopoietic progenitor cells. Rejuvenation Res.

[30] Zhu X, Gui J, Dohkan J, Cheng L, Barnes PF, Su DM (2007). Lymphohematopoietic progenitors do not have a synchronized defect with age-related thymic involution. Aging Cell.

[31] Gui J, Zhu X, Dohkan J, Cheng L, Barnes PF, Su DM (2007). The aged thymus shows normal recruitment of lymphohematopoietic progenitors but has defects in thymic epithelial cells. Int Immunol.

[32] Sutherland JS, Goldberg GL, Hammett MV, Uldrich AP, Berzins SP, Heng TS, Blazar BR, Millar JL, Malin MA, Chidgey AP, Boyd RL (2005). Activation of thymic regeneration in mice and humans following androgen blockade. J Immunol.

[33] Aspinall R, Andrew D (2001). Age-associated thymic atrophy is not associated with a deficiency in the CD44(+)CD25(-)CD3(-)CD4(-)CD8(-) thymocyte population. Cell Immunol.

[34] Thoman ML (1995). The pattern of T lymphocyte differentiation is altered during thymic involution. Mech Ageing Dev.

[35] Fowlkes BJ, Pardoll DM (1989). Molecular and cellular events of T cell development. Adv Immunol.

[36] Ballas ZK, Rasmussen W (1990). NK1.1+ thymocytes. Adult murine CD4-, CD8- thymocytes contain an NK1.1+, CD3+, CD5hi, CD44hi, TCR-V beta 8+ subset. J Immunol.

[37] Sykes M (1990). Unusual T cell populations in adult murine bone marrow. Prevalence of CD3+CD4-CD8- and alpha beta TCR+NK1.1+ cells. J Immunol.

[38] Lau LL, Spain LM (2000). Altered aging-related thymic involution in T cell receptor transgenic, MHC-deficient, and CD4-deficient mice. Mech Ageing Dev.

[39] Ohashi PS, Pircher H, Burki K, Zinkernagel RM, Hengartner H (1990). Distinct sequence of negative or positive selection implied by thymocyte T-cell receptor densities. Nature.

[40] Yeung RS, Penninger J, Mak TW (1994). T-cell development and function in gene-knockout mice. Curr Opin Immunol.

[41] Leposavic G, Pesic V, Kosec D, Radojevic K, Arsenovic-Ranin N, Pilipovic I, Perisic M, Plecas-Solarovic B (2006). Age-associated changes in CD90 expression on thymocytes and in TCR-dependent stages of thymocyte maturation in male rats. Exp Gerontol.

[42] Dion ML, Poulin JF, Bordi R, Sylvestre M, Corsini R, Kettaf N, Dalloul A, Boulassel MR, Debre P, Routy JP, Grossman Z, Sekaly RP, Cheynier R (2004). HIV infection rapidly induces and maintains a substantial suppression of thymocyte proliferation. Immunity.

[43] Hsu HC, Scott DK, Mountz JD (2005). Impaired apoptosis and immune senescence - cause or effect. Immunol Rev.

[44] Vallejo AN (2005). CD28 extinction in human T cells: altered functions and the program of T-cell senescence. Immunol Rev.

[45] Hsu HC, Zhou T, Shi J, Yang PA, Liu D, Zhang HG, Bluethmann H, Mountz JD (2001). Aged mice exhibit in vivo defective peripheral clonal deletion of D(b)/H-Y reactive CD8(+) T cells. Mech Ageing Dev.

[46] Jamieson BD, Douek DC, Killian S, Hultin LE, Scripture-Adams DD, Giorgi JV, Marelli D, Koup RA, Zack JA (1999). Generation of functional thymocytes in the human adult. Immunity.

[47] Berzins SP, Boyd RL, Miller JF (1998). The role of the thymus and recent thymic migrants in the maintenance of the adult peripheral lymphocyte pool. J Exp Med.

[48] Berzins SP, Godfrey DI, Miller JF, Boyd RL (1999). A central role for thymic emigrants in peripheral T cell homeostasis. Proc Natl Acad Sci U S A.

[49] Gray DH, Seach N, Ueno T, Milton MK, Liston A, Lew AM, Goodnow CC, Boyd RL (2006). Developmental kinetics, turnover, and stimulatory capacity of thymic epithelial cells. Blood.

[50] Min D, Panoskaltsis-Mortari A, Kuro OM, Hollander GA, Blazar BR, Weinberg KI (2007). Sustained thymopoiesis and improvement in functional immunity induced by exogenous KGF administration in murine models of aging. Blood.

[51] Palmer DB, Viney JL, Ritter MA, Hayday AC, Owen MJ (1993). Expression of the alpha beta T-cell receptor is necessary for the generation of the thymic medulla. Dev Immunol.

[52] Surh CD, Ernst B, Sprent J (1992). Growth of epithelial cells in the thymic medulla is under the control of mature T cells. J Exp Med.

[53] van Ewijk W, Hollander G, Terhorst C, Wang B (2000). Stepwise development of thymic microenvironments in vivo is regulated by thymocyte subsets. Development.

[54] Hollander GA, Wang B, Nichogiannopoulou A, Platenburg PP, van Ewijk W, Burakoff SJ, Gutierrez-Ramos JC, Terhorst C (1995). Developmental control point in induction of thymic cortex regulated by a subpopulation of prothymocytes. Nature.

[55] Boehm T, Scheu S, Pfeffer K, Bleul CC (2003). Thymic medullary epithelial cell differentiation, thymocyte emigration, and the control of autoimmunity require lympho-epithelial cross talk via LTbetaR. J Exp Med.

[56] Aw D, Taylor-Brown F, Cooper K, Palmer DB (2009). Phenotypical and morphological changes in the thymic microenvironment from ageing mice. Biogerontology.

